# Mendelian Randomization Identifies IL-4, IL-6, CCL19, and DNER as Potential Causal Inflammatory Proteins in Allergic Rhinitis: Evidence Partially Supported by Transcriptomics and Protein Interaction Analysis

**DOI:** 10.7759/cureus.92879

**Published:** 2025-09-21

**Authors:** Shiming Quan, Fuquan Zhang

**Affiliations:** 1 Otolaryngology, Beijing University of Chinese Medicine Third Affiliated Hospital, Beijing, CHN; 2 Psychiatry, The Affiliated Brain Hospital of Nanjing Medical University, Nanjing, CHN

**Keywords:** allergic rhinitis, circulating inflammatory proteins, cytokine, differential gene expression, immunotherapeutic targets, mendelian randomization

## Abstract

Background

Inflammatory proteins play a significant role in the pathogenesis of allergic rhinitis (AR), but the causal relationships remain unclear. We aimed to identify potential causal circulating inflammatory proteins contributing to the development of AR.

Methods

We employed Mendelian randomization (MR) analysis to determine the causal relationship between 91 circulating inflammatory proteins and AR. Inverse variance weighted (IVW) was the main analytic pipeline, followed by sensitivity analysis. The genome-wide association study (GWAS) summary datasets for AR and circulating inflammatory proteins were used for the analysis. The AR dataset comprised 12,240 cases and 392,069 controls, while the summary datasets for 91 plasma proteins contained 14,824 participants. Subsequent to the MR analysis, bioinformatic analysis was employed to probe differences in specific gene expression levels and to construct a network of associated proteins.

Results

The results of the MR analysis indicated that four inflammatory proteins had a causal effect on AR, including interleukin-4 (IL-4), interleukin-6 (IL-6), Chemokine (C-C motif) ligand 19 (CCL19), and Delta/Notch-like epidermal growth factor (EGF)-related receptor (DNER). Through differential gene expression analysis, IL4 mRNA expression was significantly elevated in AR patients compared to healthy controls. Protein-protein interaction (PPI) analysis via the STRING database revealed that IL-6, IL-4, and CCL19 constituted a densely connected network.

Conclusion

Our study supports the involvement of four circulating inflammatory proteins (IL-4, IL-6, CCL19, and DNER), especially IL-4, in susceptibility to AR. These findings highlight IL-4, IL-6, CCL19, and DNER as promising immunotherapeutic targets, providing mechanistic insights for developing novel diagnostics and biologic therapies for AR management.

## Introduction

Allergic rhinitis (AR) represents a globally prevalent condition, affecting 10-30% of adults and up to 40% of children [1,2]. Its clinical burden extends beyond healthcare systems to significantly impact quality of life, driven by distressing symptoms including sneezing, nasal congestion with watery discharge, and ocular pruritus [3]. Current management strategies, encompassing allergen avoidance, pharmacotherapy, and immunotherapy, rely heavily on personalized regimens to achieve effective symptom control. Multiple risk factors contribute to AR pathogenesis, such as familial predisposition, environmental allergen exposure, individual immune status, and smoking history. Given this multifactorial complexity, a comprehensive approach is essential to alleviate symptoms, improve quality of life, and halt disease progression. 

The pathogenesis of AR involves intricate immune dysregulation, with inflammatory proteins serving as central mediators in both initiating and sustaining allergic responses. Upon allergen exposure, the immune system triggers a cascade of inflammatory protein release, with interleukin-4 (IL-4), interleukin-5 (IL-5), and chemokine (C-C motif) ligand 11 (CCL11) emerging as key players. These molecules facilitate immune cell recruitment to inflamed tissues, thereby amplifying the characteristic symptoms of AR. Specifically, IL-4 promotes differentiation of naive T cells into Th2 lymphocytes, critical drivers of allergic inflammation, while IL-5 supports eosinophil survival and activation, a hallmark feature of allergic responses. CCL11 (eotaxin) further enhances eosinophil recruitment and accumulation within nasal mucosal tissues [4,5]. 

Given their central role in AR pathophysiology, targeting these inflammatory proteins has become a cornerstone of therapeutic development. Current interventions aim to modulate the levels and functions of these mediators to relieve symptoms and improve patient outcomes. Promising results from clinical trials investigating biologic therapies, including monoclonal antibodies against IL-4, IL-5, and CCL11, offer new therapeutic avenues for patients with severe, refractory AR [6]. 

Despite these advancements, critical gaps remain in understanding the causal relationships between inflammatory cytokines and AR, particularly regarding the directionality of these effects [7,8]. While cross-sectional studies face inherent limitations from confounding variables (e.g., environmental exposures, medication use) and reverse causation [7,8], Mendelian randomization (MR) analysis overcomes these through genetic instrumental variables. To address these limitations, we employed a bioinformatics approach combined with MR analysis, a method validated for robust causal inference [9-12]. Using genome-wide association study (GWAS) data for AR and 91 circulating inflammatory proteins, our study aimed to identify potential causal relationships between these proteins and AR. Unlike recent MR studies focusing on limited cytokine panels [13-15], our investigation comprehensively examines 91 inflammatory proteins quantified via the Olink Target platform [16], enabling discovery of novel associations. Circulating inflammatory protein data were derived from a large-scale GWAS (N=14,824) quantifying 91 plasma proteins using the Olink Target platform [16], providing extensive proteomic coverage. By utilizing genetic variants as instrumental variables (IVs), MR analysis mitigates confounding and reverse causation, providing more reliable evidence for inflammatory protein involvement in AR pathogenesis. Complementarily, our bioinformatics approach comprehensively evaluated the expression patterns, prognostic significance, and diagnostic utility of genes encoding these circulating inflammatory proteins in AR. Identifying causal inflammatory proteins is a pivotal step in advancing the management of AR, as it may enable the development of early diagnostic biomarkers. Furthermore, such discoveries could facilitate the design of novel biologic therapies, including monoclonal antibodies, and ultimately support the creation of personalized treatment strategies for patients with refractory forms of the condition.

## Materials and methods

Study overview

**Figure 1:**
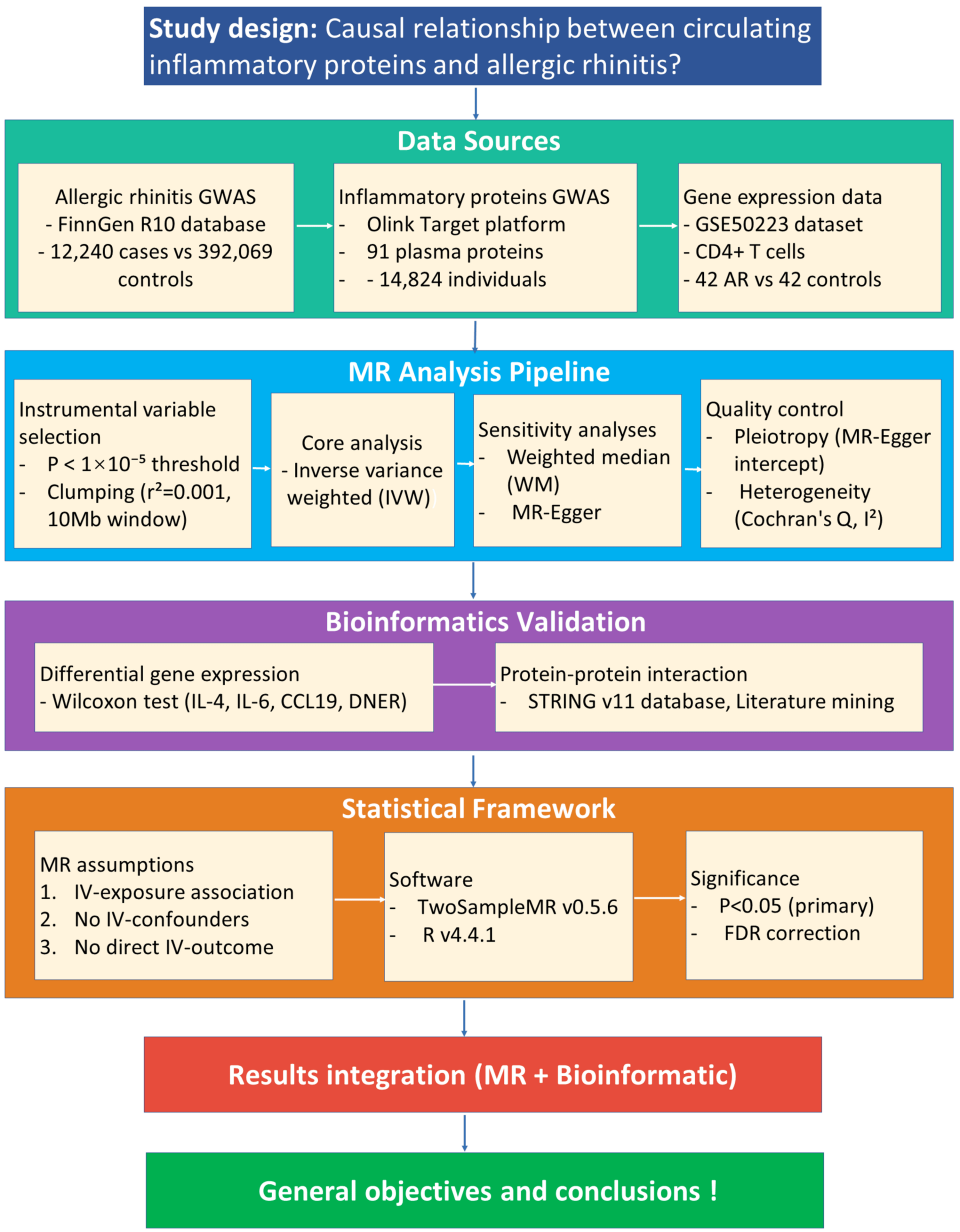
The overview of the workflow SNPs: Single-nucleotide polymorphisms; MR: Mendelian randomization; AR: allergic rhinitis; IVW: inverse variance weighting; WM: weighted median; GWAS: genome-wide association study; PPI: protein-protein interaction; IVs: instrumental variables

We designed a two-stage investigation. First, MR was used to establish causal links. Following this, bioinformatic analyses explored differential gene expression and protein-protein interaction (PPI) networks. The analytical framework is depicted in Figure 1.

MR study design

**Figure 2:**
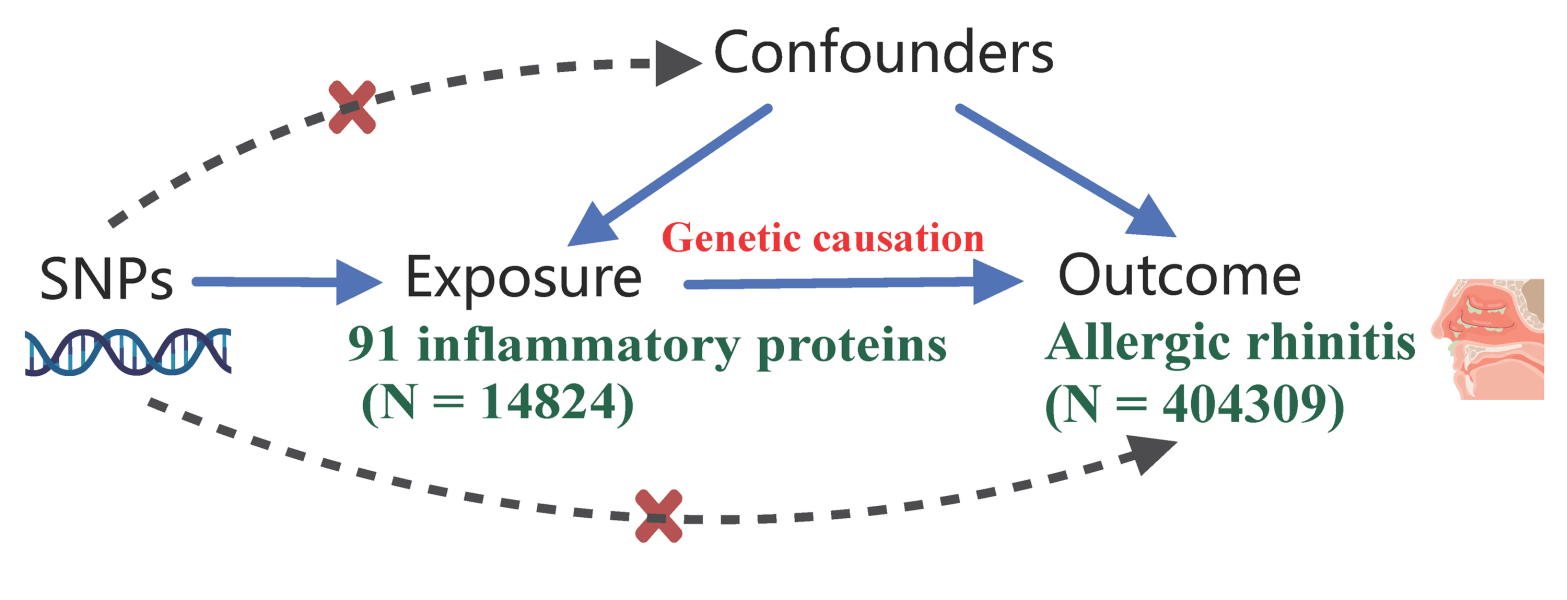
The overview of the MR study AR data: FinnGen R10 (12,240 cases/392,069 controls). Inflammatory protein data: Olink GWAS (14,824 individuals). MR: Mendelian randomization

MR was applied to evaluate causal associations between 91 circulating inflammatory proteins and AR. Inverse variance weighting (IVW) served as the primary method, with sensitivity analyses conducted using weighted median (WM) and MR-Egger approaches. All analyses utilized genome-wide association study (GWAS) summary datasets for AR and circulating inflammatory proteins, as outlined in Figure 2. 

Data source description

GWAS summary data were acquired from public repositories. AR data included 12,240 cases and 392,069 controls from FinnGen R10 (Study ID: finn-b-J10_ALLERGIC_RHINITIS; https://finngen.gitbook.io/documentation/v/r10/) [17]. FinnGen R10 was selected for AR GWAS data due to its large sample size (over 400,000 total participants) and well-characterized AR cases, which enhances statistical power for detecting associations; additionally, it provides comprehensive genetic and phenotypic data specific to European populations, ensuring reliability for causal inference in our study. Circulating inflammatory protein data (91 proteins) came from the Olink Target GWAS (accession: GCST90274758-GCST90274848; https://www.ebi.ac.uk/gwas/) [16]. The Olink Target platform was chosen for inflammatory protein GWAS data because it enables high-sensitivity quantification of 91 clinically relevant inflammatory proteins in a large cohort (N=14,824), covering key mediators of immune and inflammatory responses-critical for comprehensively evaluating potential causal proteins in AR pathogenesis. Original studies were ethically approved; our secondary analysis of existing data required no additional ethical clearance.

MR analysis of circulating inflammatory proteins and AR

MR relies on IVs meeting three criteria: (i) association with exposure; (ii) no association with exposure-outcome confounders; (iii) no direct effect on outcome beyond exposure. To analyze the causal relationship between circulating inflammatory proteins and AR. We used three models from the TwoSampleMR package (v0.5.6) [18], selected for their robust pleiotropy-correction methods. Primary analysis employed IVW, which weights IV estimates to reduce random error under the assumption of valid IVs with exposure-mediated effects on outcome. Sensitivity analyses used WM (prioritizing precise IVs despite potential invalidity) and MR-Egger (detecting and correcting invalid IV biases).

Differential gene expression analysis

Differential gene expression analysis compares transcriptional profiles across sample groups (e.g., disease vs. control), using high-throughput sequencing or microarrays to identify expression changes linked to physiological or pathological states. This informs disease mechanisms, biomarker discovery, and therapeutic target identification [19,20]. 

Gene expression data (GSE50223) were retrieved from the NCBI Gene Expression Omnibus (https://www.ncbi.nlm.nih.gov/geo/query/acc.cgi) [21], including 42 control and 42 AR CD4^+^ T cell samples. Wilcoxon tests compared IL-6, IL-4, DNER and CCL19 gene expression between groups to identify significant alterations. By comparing the distribution of these reads in different samples, it can be found that the expression of some genes in specific samples significantly increased or decreased.

PPI analysis

To explore mechanisms linking circulating inflammatory proteins to AR, we mined literature and analyzed PPIs among relevant genes. PPIs were analyzed using STRING v11.0 (https://string-db.org) with medium confidence (combined score >0.7). Literature mining was conducted separately via PubMed. This tool integrates known/predicted interactions from multiple databases, with a user-friendly interface for network visualization. Analyses identified key molecular interactions/pathways, enhancing understanding of AR pathogenesis and therapeutic target discovery [22].

Statistical analyses

All analyses used R v4.4.1 with TwoSampleMR and STRINGdb packages. IVs were genome-wide significant SNPs (P < 1×10^-5^) associated with exposure phenotypes. The cutoff of P < 1×10^-5^ for SNP selection was adopted based on standard practices in MR studies for inflammatory proteins; this threshold balances statistical stringency (to ensure SNPs are robustly associated with exposure) and retention of sufficient IVs for reliable causal estimation. SNPs within 10 Mb of each other were excluded using clumping (r^2^ = 0.001) to minimize genetic correlation, ensuring IV independence and MR result robustness. The clumping parameters (10 Mb window, r^2^ = 0.001) were chosen to reduce linkage disequilibrium (LD) between SNPs, as recommended by the TwoSampleMR package guidelines. The low r^2^ threshold ensures minimal residual LD, preserving IV independence and reducing bias in causal estimates.

Sensitivity analyses used MR-Egger intercept to detect horizontal pleiotropy (non-zero intercept indicates IV effects independent of exposure). Heterogeneity was assessed via Cochran's Q test and I^2^ statistic, with significance at P < 0.05 and I^2^ > 0.25 [23].

## Results

Causal effects of circulating inflammatory proteins on AR

**Figure 3:**
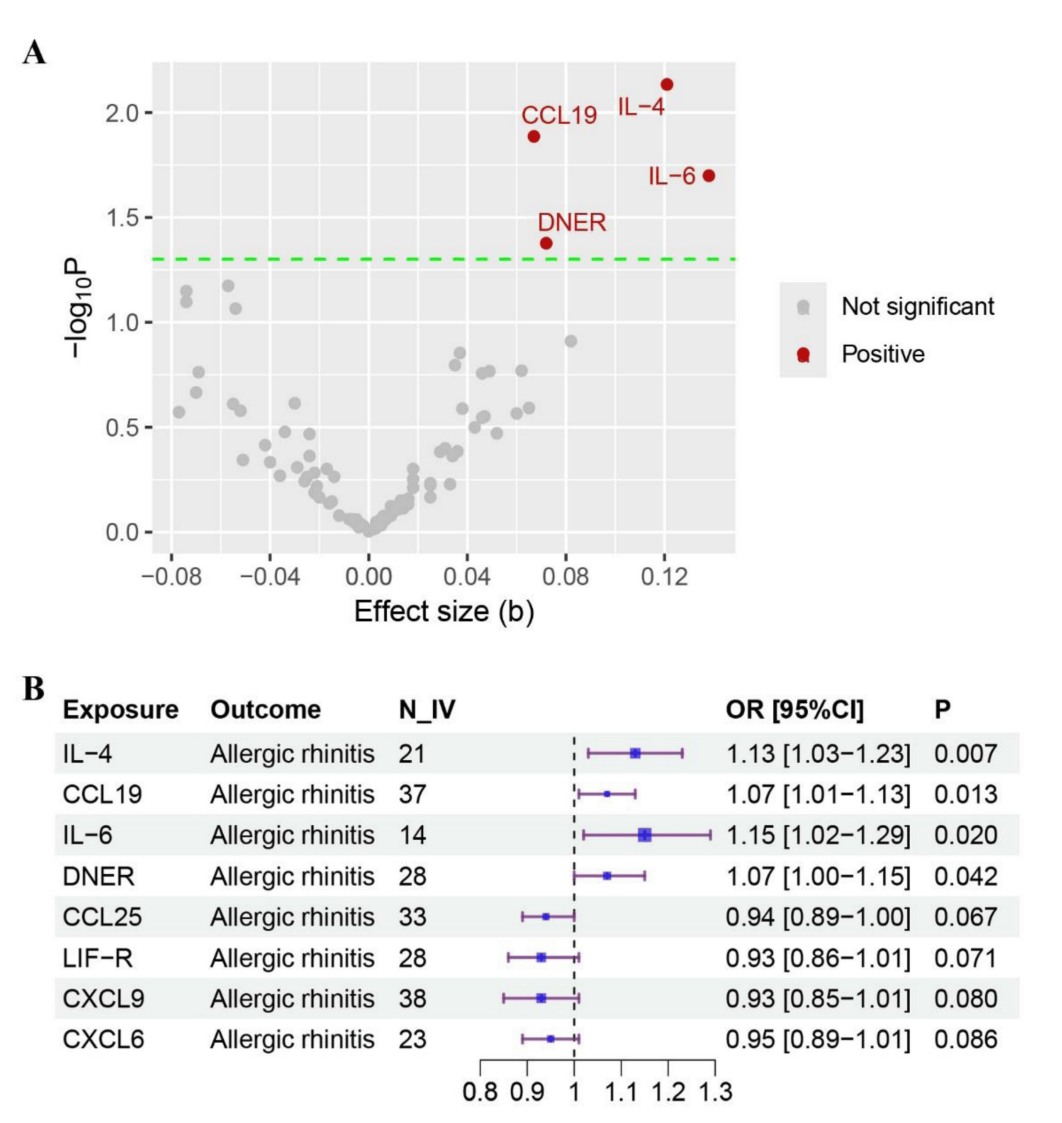
MR analysis of inflammatory proteins on AR risk A: Volcano plot displaying causal effect estimates for plasma inflammatory proteins. Points: Proteins with significant positive causal effects (red; FDR < 0.05) and non-significant associations (gray). X-axis: Effect size per standard deviation increase in protein concentration (positive values: risk-increasing effects; negative values: protective effects). Y-axis: Statistical significance (–log₁₀-transformed p-values). Threshold: Dashed horizontal line indicates significance (p = 0.05). B: Forest plot of causal effects of inflammatory proteins on AR risk using MR. Odds ratios (ORs) with 95% confidence intervals represent the causal effect of a one-standard deviation increase in circulating protein levels on AR susceptibility. Significant associations (p < 0.05) are highlighted: IL-4，CCL19, IL-6 and DNER. Analyses used inverse-variance weighted MR with N_IV (number of genetic instruments) ranging from 14–38 per protein. The vertical line (OR = 1) indicates no effect. MR: Mendelian randomization; AR: allergic rhinitis

**Figure 4:**
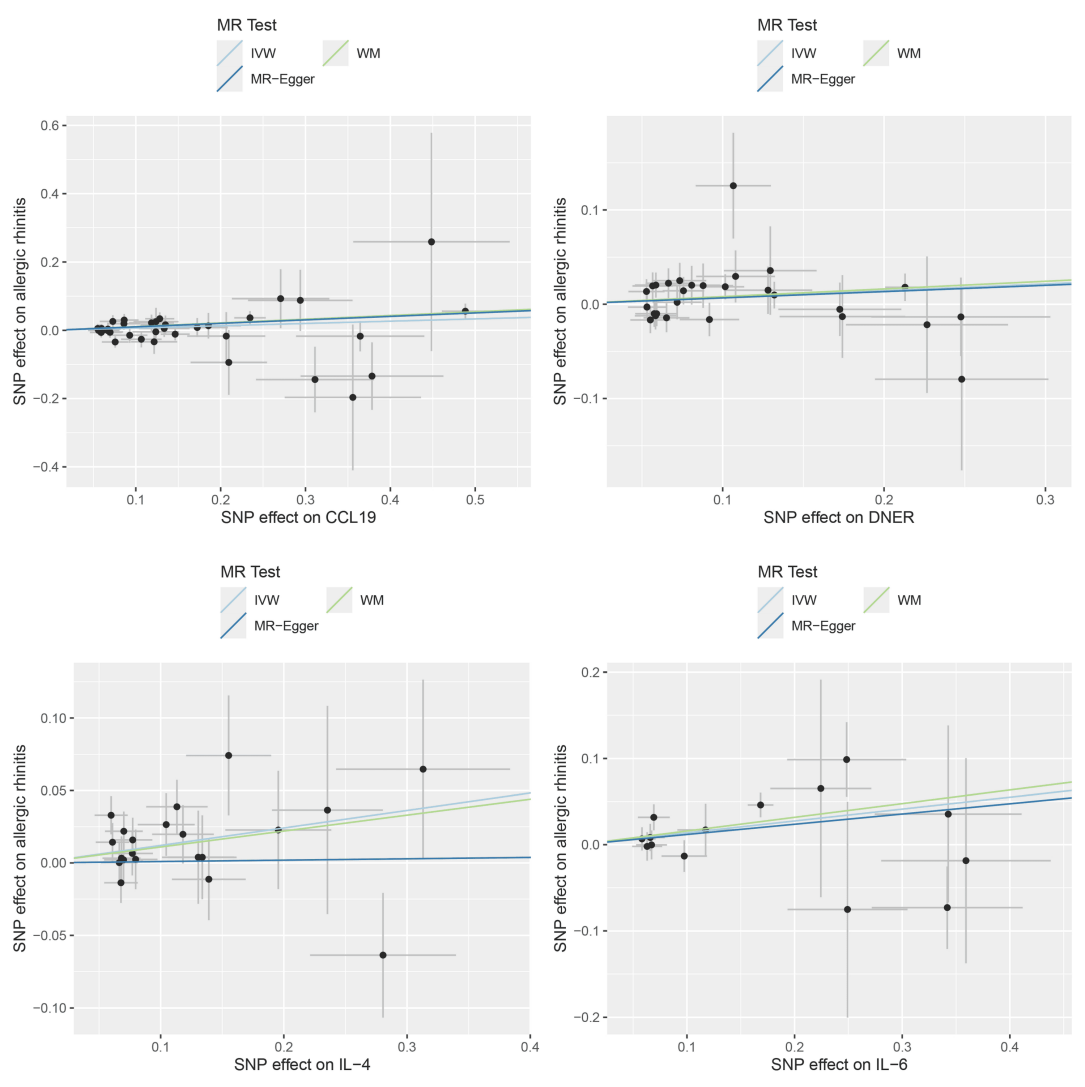
Causal effects of four circulating inflammatory proteins on AR The trait on the x-axis denotes the exposure, the trait on the y-axis denotes the outcomes, and each cross point represents an instrumental variant. The lines denote the effect sizes (b) of the MR analysis. MR: Mendelian randomization; AR: allergic rhinitis

Of 91 proteins analyzed, four met significance thresholds (IL-4, IL-6, CCL19, DNER). The remaining 87 showed no significant associations. A total of 21, 14, 37, and 28 IVs were yielded for IL-6, IL-4, CCL19, and DNER, respectively. The MR analysis identified four inflammatory proteins are associated with an increased risk of AR, including IL-4 (OR: 1.13, 95% confidence interval (CI): 1.03-1.23, P = 0.007), IL-6 (OR: 1.15, 95% CI: 1.02-1.29, P = 0.020), CCL19 (OR: 1.07, 95% CI: 1.01-1.13, P = 0.013), and DNER (OR: 1.07, 95% CI: 1.00-1.15, P = 0.042) (Table 1 and Figures 3, 4). Importantly, all four inflammatory proteins were associated with an increased risk of AR.

**Table 1:** Causal effects of circulating inflammatory proteins on AR OR: odds ratio; CI: confidence interval; N_IV: number of instrumental variables; Q_P: Cochran’s P-value of heterogeneity analysis; AR: allergic rhinitis

Exposure	N_IV		OR [95%CI]	Q_P		I^2^	P_pleiotropy			P
IL-4		21	1.13 [1.03-1.23]		0.508	-0.041		0.300	0.007	
CCL19		37	1.07 [1.01-1.13]		0.64	-0.111		0.305	0.013	
IL-6		14	1.15 [1.02-1.29]		0.185	0.25		0.857	0.020	
DNER		28	1.07 [1.00-1.15]		0.61	-0.108		0.941	0.042	

Sensitivity analysis

In the sensitivity analysis, both MR-Egger and WM methods yielded consistent results in terms of the direction of causal effects (Table 1 and Figures 3,4). No significant horizontal pleiotropy was detected in the MR-Egger regression analysis (P > 0.05). Cochran's Q test and the I^2^ statistic did not support the heterogeneity in the MR estimates (P > 0.05).

Differential gene expression analysis

**Figure 5:**
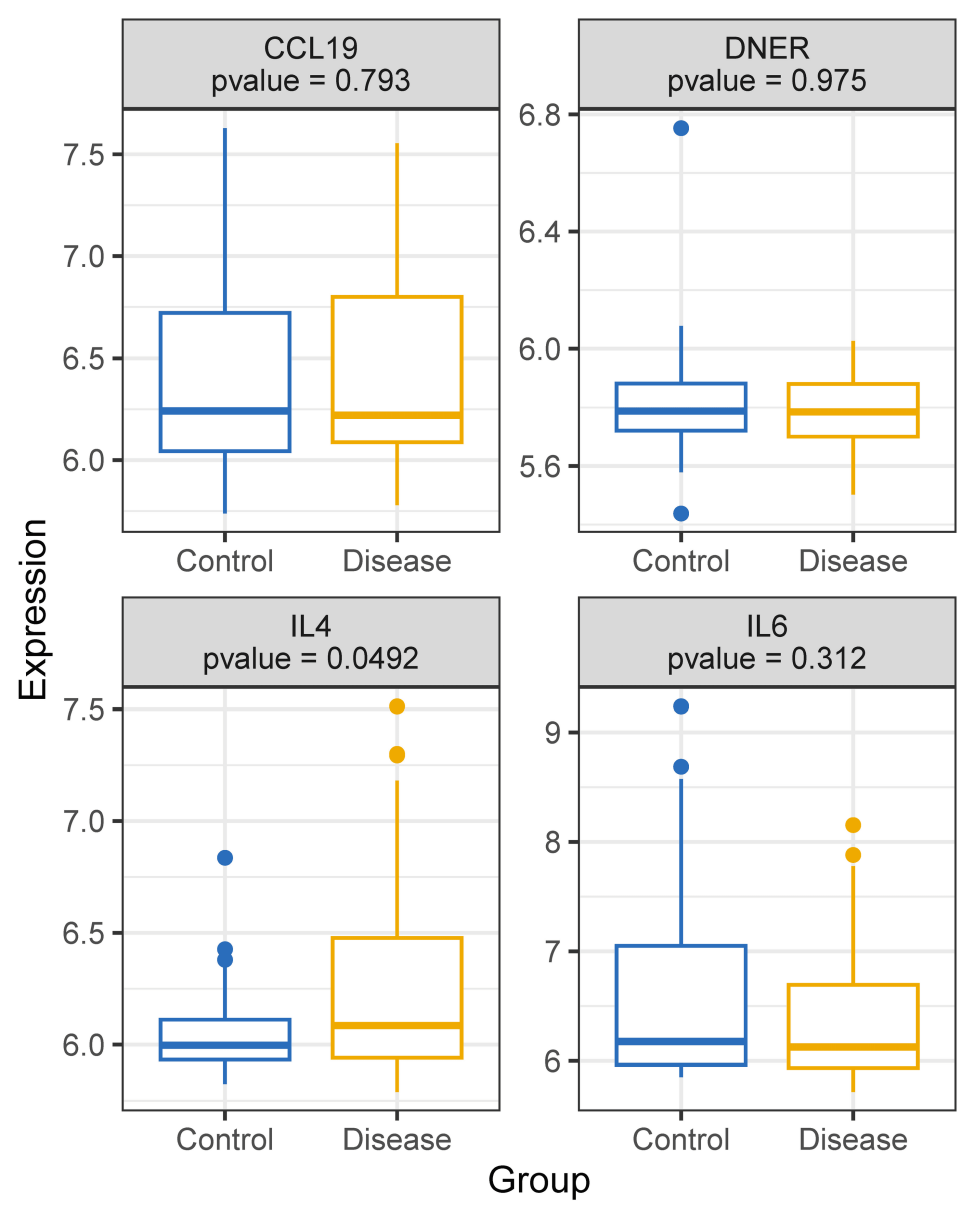
Differential gene expression analysis in allergic rhinitis Boxplots comparing gene expression levels of CCL19, IL4, IL6, and DNER between healthy controls and allergic rhinitis (AR) patients (GSE50223 dataset). P-values from differential expression analysis are shown for each gene. IL4 expression is significantly elevated in AR patients versus controls (p = 0.0492), whereas CCL19, IL6, and DNER show no significant differences. Boxes indicate interquartile ranges (IQR), central lines represent medians, and whiskers extend to 1.5× IQR.

Through gene difference analysis, researchers found that the expression level of IL-4 gene in patients with AR was significantly higher than that in healthy people, as shown in Figure 5. 

PPI analysis

**Figure 6:**
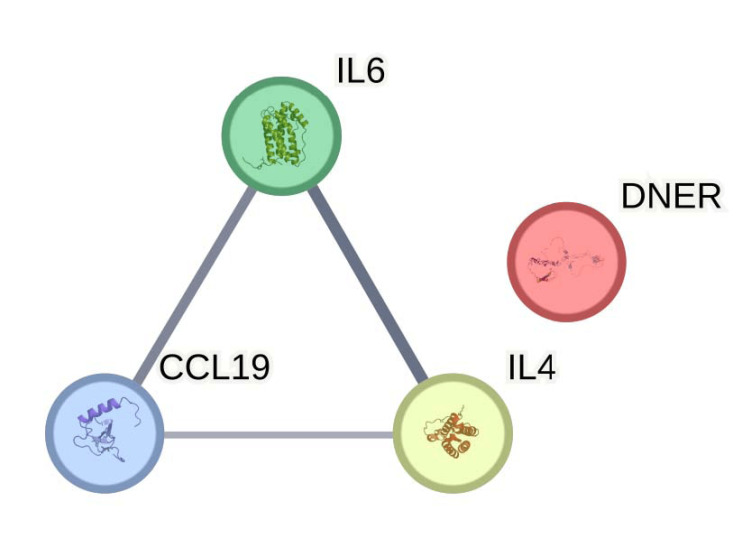
String analysis of protein-protein interaction Protein−protein interactions among IL-6, IL-4, CCL-19 and DNER, the 4 AR-promoting genes. Line sizes are proportional to the combined scores of the interactions. No clear effect relationship was found between DNER and other three proteins. IL-6: Interleukin-6; IL-4: Interleukin-4; CCL-19: Chemokine (C-C motif) ligand 19; DNER: Delta/Notch-like epidermal growth factor (EGF)-related receptor

Interactions were identified using STRING. PPI analysis revealed that IL-6, IL-4 and CCL19 constituted a densely connected network (Figure 6). No clear effect relationship was found between DNER and other three proteins, IL-6, IL-4, and CCL-19. These proteins exhibited significant interaction scores.

## Discussion

The main strength of the study lies in its use of MR analysis, which is less susceptible to causality-related errors often seen in traditional observational studies. These errors typically arise from confounding factors and reverse causation. The study leveraged the largest accessible GWAS datasets to date to establish a causal link between circulating inflammatory proteins and AR. The MR analysis corroborates prior links between IL-6, CCL19, DNER, and AR while newly implicating IL-4 [13]. This expands the mechanistic understanding beyond Th2 cytokines. As shown in Figure 3, the key Inflammatory factors, such as IL-6, IL-4, CCL19, and DNER, play a vital role in the pathophysiology of AR. These kinds of proteins, such as cytokines and chemokines, are released by immune cells in response to allergen exposure, leading to the recruitment of more immune cells to the site of inflammation. In the progression of AR, there are a series of hot and difficult topics [24], to reveal the mechanism of circulating inflammatory proteins in the initiation and cascade amplification of allergic reactions in AR, to manipulate this inflammatory process to promote the positive transformation of inflammation, and to alleviate or avoid a series of pathological and physiological reactions caused by specific circulating inflammatory proteins, such as IL-6, IL-4, CCL19 and other potential unknown cytokines [4]. 

IL-6 is a pro-inflammatory cytokine that plays a significant role in the inflammatory response associated with AR. Elevated levels of IL-6 have been found in the nasal mucosa of individuals with AR, indicating its involvement in the inflammatory response [25]. Additionally, IL-6 contributes to the recruitment of eosinophils and mast cells to the nasal mucosa, further exacerbating the allergic response in individuals with AR. IL-6 can be influenced by both therapeutic interventions and gene regulation [26]. Therefore, the association between IL-6 and AR extends beyond local inflammation, offering potential targets for intervention and treatment strategies.

IL-4 is a crucial cytokine in the context of AR, as it drives the activation and differentiation of Th2 cells, pivotal players in the allergic response [4]. Moreover, IL-4 is instrumental in the generation of IgE antibodies by B cells, thus intensifying the allergic reaction within the nasal mucosa. This elevation in IL-4 levels in AR is associated with allergic inflammation, primarily through the stimulation of IgE production and the recruitment and activation of eosinophils, the key effector cells in the inflammatory cascade characteristic of AR [27]. Thus, we can monitor the expression level of IL-4 and assess the serious degree in the evolution and outcome of annoying AR.

Through gene difference analysis, we found that the expression level of the IL-4 gene in patients with AR was significantly higher than that in healthy people (Figure 5). This may be because, in the process of AR, the immune system of the body is activated by allergens, resulting in the enhancement of the Th2 cell-dominated immune response. The differential expression of the IL-4 gene can be an important link in the pathogenesis of AR. It plays a key role in the pathological process of AR by regulating the production of IgE by B cells and activating inflammatory cells to release mediators. This gene expression difference provides a potential target for the diagnosis, treatment and prevention of AR. For example, monoclonal antibody drugs against IL-4 can be used to treat allergic rhinitis and reduce allergic symptoms by blocking the effect of IL-4. Dupilumab is a fully human IgG4 monoclonal antibody that targets the interleukin-4 receptor α-subunit (IL-4Rα). It dual-blocks signaling of IL-4 and IL-13, thereby down-regulating type-2 inflammatory pathways. The data show that dupilumab significantly improved all clinical and biomarker endpoints and reduced systemic corticosteroid use or need for sinonasal surgery in severe chronic rhinosinusitis with nasal polyps (CRSwNP) over 24 weeks, with these benefits and an identical safety profile observed whether or not patients had concomitant AR, confirming dupilumab’s consistent efficacy irrespective of AR status [28].

CCL19, also known as macrophage inflammatory protein-3 beta (MIP-3 beta), is a chemokine crucial in the pathogenesis of AR by orchestrating the recruitment and activation of immune cells at the nasal mucosa [29]. Elevated CCL19 levels in AR lead to the attraction of dendritic cells and T cells, intensifying nasal mucosa inflammation and exacerbating allergic symptoms. Additionally, CCL19 inhibitors may disrupt immune cell recruitment [29]. CCL19's involvement in promoting Th2 responses and allergic reactions suggests a potential therapeutic avenue through IL-10-expressing regulatory T cells for monitoring, treating and managing AR. 

We have uncovered a newly identified association between DNER and AR. DNER, a transmembrane protein featuring EGF-like repeats, has been implicated in cell-cell signaling and adhesion processes. The findings suggest that DNER may play a role in the pathogenesis of AR, potentially by influencing immune cell interactions or modulating inflammatory responses. This discovery not only broadens our understanding of the complex immunological landscape of AR but also opens up new avenues for therapeutic intervention. DNER represents a novel target for biologics development. Further research is warranted to delineate the precise molecular mechanisms underlying this association and to explore the potential of DNER-targeted therapies in the management of AR. As the finding of a causal relationship between DNER and AR and asthma [13,30]. 

PPI analysis using the STRING database revealed that IL-6, IL-4 and CCL19 formed a tightly interconnected network (Figure 6). No clear effect relationship was found between DNER and other three proteins, IL-4, IL-6, and CCL-19. These proteins exhibited significant interaction scores, indicating their potential synergistic effects in the pathophysiological processes of AR. The network analysis suggested that these key inflammatory mediators might collectively contribute to the development and progression of AR symptoms, highlighting them as potential targets for therapeutic intervention in the following research [6].

Certainly, the study has several limitations, experimental validation is needed to confirm mechanistic roles [18]. First, only a limited number of inflammatory cytokines were involved in this study. Second, it is important to note that the pro-inflammatory responses induced and regulated by these cytokines represent only a portion of the intricate pathogenesis of AR. Lastly, our study revealed several circulating inflammatory proteins causally associated with AR, but the detailed physiological mechanisms require further experimental investigation [6,7].

Additionally, common SNPs may not fully account for diseases like AR, which has a multi-genetic basis. Also, coding genes make up just 1-2% of the human genome. This research didn't explore SNPs in non-coding regions, but if these SNPs are in functionally critical non-coding areas, they might influence the disease. In addition, we must be aware of whether the geographical nature of the genetic data is widely representative [18]. Future research should continue to explore the underlying mechanisms and validate these findings in diverse populations, ultimately leading to more effective and trustworthy results and conclusions.

## Conclusions

MR evidence links specific circulating inflammatory proteins to AR in a causal manner, clarifying the contribution of systemic inflammation to disease pathogenesis. Among the implicated mediators, IL-4 exerts the most pronounced effect, while IL-6, CCL19, and DNER display additional, albeit weaker, influences. These proteins emerge as quantifiable therapeutic targets that can guide clinical monitoring, individualized treatment, and drug development for AR.

## Appendices

**Table 2:** Supplementary table listing all 91 inflammatory proteins tested

Exposure	Outcome	N_IV	P_IV	b (se)	OR [95%CI]	Q_P	I2	Egger_intercept	P_pleiotropy	P
IL-4	Allergic rhinitis	21	1.00E-05	0.121 (0.045)	1.13 [1.03-1.23]	0.508	-0.041	0.011	0.3	7.35E-03
CCL19	Allergic rhinitis	37	1.00E-05	0.067 (0.027)	1.07 [1.01-1.13]	0.64	-0.111	-0.006	0.305	0.013
IL-6	Allergic rhinitis	14	1.00E-05	0.138 (0.059)	1.15 [1.02-1.29]	0.185	0.25	0.002	0.857	0.02
DNER	Allergic rhinitis	28	1.00E-05	0.072 (0.035)	1.07 [1.00-1.15]	0.61	-0.108	0.001	0.941	0.042
CCL25	Allergic rhinitis	33	1.00E-05	-0.057 (0.031)	0.94 [0.89-1.00]	3.19E-03	0.449	0	0.946	0.067
LIF-R	Allergic rhinitis	28	1.00E-05	-0.074 (0.041)	0.93 [0.86-1.01]	0.012	0.417	0.002	0.858	0.071
CXCL9	Allergic rhinitis	38	1.00E-05	-0.074 (0.042)	0.93 [0.85-1.01]	0.035	0.314	0.001	0.923	0.08
CXCL6	Allergic rhinitis	23	1.00E-05	-0.054 (0.032)	0.95 [0.89-1.01]	0.175	0.215	-0.008	0.259	0.086
SIRT2	Allergic rhinitis	21	1.00E-05	0.082 (0.053)	1.09 [0.98-1.21]	0.071	0.332	-0.004	0.721	0.123
ADA	Allergic rhinitis	24	1.00E-05	0.037 (0.025)	1.04 [0.99-1.09]	0.642	-0.15	-0.004	0.424	0.14
IL-18R1	Allergic rhinitis	38	1.00E-05	0.035 (0.025)	1.04 [0.99-1.09]	0.02	0.349	0.013	0.044	0.16
CCL7	Allergic rhinitis	26	1.00E-05	0.062 (0.045)	1.06 [0.97-1.16]	0.027	0.381	0.019	0.113	0.17
FGF-19	Allergic rhinitis	32	1.00E-05	0.049 (0.036)	1.05 [0.98-1.13]	0.812	-0.293	0.003	0.789	0.171
IL-1 alpha	Allergic rhinitis	24	1.00E-05	-0.069 (0.050)	0.93 [0.85-1.03]	0.027	0.391	0.013	0.312	0.173
CXCL5	Allergic rhinitis	22	1.00E-05	0.046 (0.034)	1.05 [0.98-1.12]	0.088	0.305	-0.003	0.725	0.175
IL-5	Allergic rhinitis	23	1.00E-05	-0.070 (0.056)	0.93 [0.84-1.04]	0.01	0.454	-0.021	0.089	0.216
TRAIL	Allergic rhinitis	38	1.00E-05	-0.030 (0.025)	0.97 [0.92-1.02]	0.174	0.176	-0.012	0.066	0.243
CD5	Allergic rhinitis	32	1.00E-05	-0.055 (0.047)	0.95 [0.86-1.04]	3.10E-03	0.455	0.016	0.17	0.245
MMP-10	Allergic rhinitis	24	1.00E-05	0.065 (0.058)	1.07 [0.95-1.20]	3.90E-05	0.616	0.013	0.301	0.256
CCL13	Allergic rhinitis	31	1.00E-05	0.038 (0.034)	1.04 [0.97-1.11]	0.081	0.274	0.002	0.842	0.258
IL-10	Allergic rhinitis	31	1.00E-05	-0.052 (0.046)	0.95 [0.87-1.04]	6.46E-03	0.43	0.008	0.421	0.264
TGF-alpha	Allergic rhinitis	27	1.00E-05	-0.077 (0.069)	0.93 [0.81-1.06]	4.95E-06	0.633	-0.017	0.274	0.268
IL-7	Allergic rhinitis	25	1.00E-05	0.060 (0.055)	1.06 [0.95-1.18]	0.122	0.255	0.018	0.158	0.272
IL-17C	Allergic rhinitis	36	1.00E-05	0.047 (0.044)	1.05 [0.96-1.14]	0.049	0.299	0.003	0.817	0.281
CXCL11	Allergic rhinitis	37	1.00E-05	0.046 (0.043)	1.05 [0.96-1.14]	0.024	0.342	0.005	0.665	0.285
HGF	Allergic rhinitis	32	1.00E-05	0.043 (0.043)	1.04 [0.96-1.14]	0.47	-0.003	0.008	0.401	0.317
IL-13	Allergic rhinitis	25	1.00E-05	-0.034 (0.035)	0.97 [0.90-1.04]	0.442	0.014	-0.005	0.468	0.333
CSF-1	Allergic rhinitis	29	1.00E-05	0.052 (0.055)	1.05 [0.95-1.17]	7.10E-04	0.518	0.019	0.115	0.338
SCF	Allergic rhinitis	45	1.00E-05	-0.024 (0.025)	0.98 [0.93-1.03]	0.584	-0.063	0	0.92	0.341
STAMPB	Allergic rhinitis	22	1.00E-05	-0.042 (0.048)	0.96 [0.87-1.05]	0.393	0.05	-0.003	0.789	0.385
OPG	Allergic rhinitis	30	1.00E-05	0.031 (0.036)	1.03 [0.96-1.11]	0.518	-0.036	0.007	0.38	0.399
CXCL10	Allergic rhinitis	34	1.00E-05	0.036 (0.044)	1.04 [0.95-1.13]	9.28E-03	0.401	-0.001	0.905	0.412
TWEAK	Allergic rhinitis	43	1.00E-05	0.029 (0.035)	1.03 [0.96-1.10]	0.072	0.251	-0.005	0.503	0.414
CASP-8	Allergic rhinitis	24	1.00E-05	0.034 (0.044)	1.03 [0.95-1.13]	0.314	0.106	-0.01	0.265	0.434
IL-15RA	Allergic rhinitis	23	1.00E-05	-0.024 (0.030)	0.98 [0.92-1.04]	0.125	0.26	-0.003	0.677	0.434
IL-22RA1	Allergic rhinitis	21	1.00E-05	-0.051 (0.068)	0.95 [0.83-1.09]	0.026	0.413	0.02	0.199	0.453
Beta-NGF	Allergic rhinitis	33	1.00E-05	-0.040 (0.055)	0.96 [0.86-1.07]	3.57E-03	0.445	0.012	0.346	0.464
hGDNF	Allergic rhinitis	24	1.00E-05	-0.029 (0.043)	0.97 [0.89-1.06]	0.135	0.246	-0.002	0.804	0.491
IL10RB	Allergic rhinitis	29	1.00E-05	0.018 (0.026)	1.02 [0.97-1.07]	0.208	0.171	0.006	0.298	0.5
TNFB	Allergic rhinitis	41	1.00E-05	-0.017 (0.025)	0.98 [0.94-1.03]	0.033	0.31	0.009	0.197	0.5
VEGFA	Allergic rhinitis	34	1.00E-05	-0.022 (0.034)	0.98 [0.91-1.05]	0.013	0.386	0.01	0.227	0.522
MIP-1 alpha	Allergic rhinitis	22	1.00E-05	-0.036 (0.059)	0.96 [0.86-1.08]	1.85E-08	0.73	-0.019	0.121	0.539
CST5	Allergic rhinitis	48	1.00E-05	-0.014 (0.023)	0.99 [0.94-1.03]	0.378	0.048	0.006	0.154	0.545
CCL20	Allergic rhinitis	33	1.00E-05	-0.025 (0.041)	0.98 [0.90-1.06]	0.339	0.078	-0.004	0.701	0.546
CCL2	Allergic rhinitis	31	1.00E-05	0.018 (0.030)	1.02 [0.96-1.08]	0.25	0.138	0.008	0.244	0.559
SLAMF1	Allergic rhinitis	38	1.00E-05	-0.026 (0.045)	0.97 [0.89-1.07]	2.22E-04	0.506	-0.002	0.801	0.572
TGF-beta-1	Allergic rhinitis	30	1.00E-05	0.025 (0.046)	1.03 [0.94-1.12]	0.023	0.371	0.003	0.721	0.585
IFN-gamma	Allergic rhinitis	20	1.00E-05	0.033 (0.062)	1.03 [0.92-1.17]	7.49E-03	0.489	-0.001	0.95	0.591
4EBP1	Allergic rhinitis	18	1.00E-05	0.025 (0.047)	1.03 [0.93-1.12]	0.472	-0.016	0.007	0.524	0.598
CD244	Allergic rhinitis	33	1.00E-05	-0.021 (0.041)	0.98 [0.90-1.06]	6.89E-03	0.419	0.005	0.552	0.605
NRTN	Allergic rhinitis	25	1.00E-05	0.018 (0.036)	1.02 [0.95-1.09]	0.688	-0.191	0.006	0.438	0.614
OSM	Allergic rhinitis	25	1.00E-05	-0.022 (0.048)	0.98 [0.89-1.08]	0.058	0.329	-0.009	0.398	0.648
AXIN1	Allergic rhinitis	13	1.00E-05	0.025 (0.060)	1.02 [0.91-1.15]	0.518	-0.078	-0.019	0.214	0.682
FGF-21	Allergic rhinitis	26	1.00E-05	-0.020 (0.049)	0.98 [0.89-1.08]	0.012	0.425	-0.011	0.319	0.683
IL-8	Allergic rhinitis	29	1.00E-05	0.016 (0.040)	1.02 [0.94-1.10]	0.944	-0.627	-0.007	0.35	0.693
IL-2RB	Allergic rhinitis	23	1.00E-05	0.016 (0.041)	1.02 [0.94-1.10]	0.669	-0.182	-0.004	0.717	0.7
CD40	Allergic rhinitis	24	1.00E-05	0.013 (0.035)	1.01 [0.95-1.09]	0.032	0.381	0.008	0.265	0.708
IL-17A	Allergic rhinitis	22	1.00E-05	-0.015 (0.041)	0.99 [0.91-1.07]	0.399	0.046	-0.003	0.752	0.715
LIF	Allergic rhinitis	24	1.00E-05	-0.016 (0.045)	0.98 [0.90-1.08]	0.506	-0.035	0.006	0.522	0.729
FGF-23	Allergic rhinitis	28	1.00E-05	0.016 (0.049)	1.02 [0.92-1.12]	0.094	0.272	-0.009	0.407	0.736
CDCP1	Allergic rhinitis	36	1.00E-05	0.009 (0.028)	1.01 [0.96-1.06]	0.37	0.058	0.008	0.193	0.753
IL-10RA	Allergic rhinitis	19	1.00E-05	0.014 (0.046)	1.01 [0.93-1.11]	0.224	0.189	0.009	0.298	0.769
NT-3	Allergic rhinitis	32	1.00E-05	0.014 (0.048)	1.01 [0.92-1.11]	0.014	0.39	0.006	0.532	0.769
EN-RAGE	Allergic rhinitis	23	1.00E-05	0.012 (0.044)	1.01 [0.93-1.10]	0.233	0.168	0.001	0.932	0.778
TNF	Allergic rhinitis	29	1.00E-05	0.010 (0.039)	1.01 [0.94-1.09]	0.335	0.085	-0.006	0.427	0.797
IL-20	Allergic rhinitis	24	1.00E-05	-0.012 (0.055)	0.99 [0.89-1.10]	0.054	0.339	-0.009	0.569	0.834
PD-L1	Allergic rhinitis	30	1.00E-05	0.009 (0.042)	1.01 [0.93-1.09]	0.285	0.117	-0.015	0.081	0.836
FIt3L	Allergic rhinitis	47	1.00E-05	0.006 (0.031)	1.01 [0.95-1.07]	0.108	0.209	0.005	0.432	0.839
CCL28	Allergic rhinitis	38	1.00E-05	0.007 (0.044)	1.01 [0.92-1.10]	0.1	0.235	-0.002	0.781	0.866
IL-20RA	Allergic rhinitis	21	1.00E-05	-0.008 (0.049)	0.99 [0.90-1.09]	0.182	0.217	-0.014	0.279	0.867
CCL4	Allergic rhinitis	30	1.00E-05	0.006 (0.037)	1.01 [0.94-1.08]	2.20E-06	0.629	-0.012	0.206	0.868
CXCL1	Allergic rhinitis	26	1.00E-05	-0.007 (0.041)	0.99 [0.92-1.08]	0.093	0.281	-0.001	0.898	0.868
TRANCE	Allergic rhinitis	51	1.00E-05	0.005 (0.031)	1.01 [0.95-1.07]	0.023	0.305	-0.007	0.312	0.869
IL-18	Allergic rhinitis	31	1.00E-05	-0.005 (0.033)	0.99 [0.93-1.06]	0.46	0.0035	0.002	0.769	0.871
uPA	Allergic rhinitis	44	1.00E-05	0.005 (0.035)	1.01 [0.94-1.08]	0.028	0.311	-0.001	0.938	0.875
IL-12B	Allergic rhinitis	37	1.00E-05	0.005 (0.033)	1.00 [0.94-1.07]	2.83E-07	0.623	0.005	0.532	0.884
MMP-1	Allergic rhinitis	26	1.00E-05	0.006 (0.039)	1.01 [0.93-1.08]	0.336	0.088	-0.008	0.31	0.887
CD6	Allergic rhinitis	27	1.00E-05	0.004 (0.031)	1.00 [0.95-1.07]	0.03	0.368	-0.004	0.64	0.888
TSLP	Allergic rhinitis	24	1.00E-05	-0.006 (0.043)	0.99 [0.91-1.08]	0.597	-0.109	-0.007	0.542	0.895
FGF-5	Allergic rhinitis	34	1.00E-05	0.003 (0.021)	1.00 [0.96-1.05]	0.652	-0.126	0.005	0.376	0.897
CCL11	Allergic rhinitis	29	1.00E-05	-0.005 (0.046)	1.00 [0.91-1.09]	9.14E-03	0.424	0.012	0.241	0.913
ST1A1	Allergic rhinitis	33	1.00E-05	-0.003 (0.031)	1.00 [0.94-1.06]	0.264	0.125	-0.008	0.327	0.921
CCL23	Allergic rhinitis	32	1.00E-05	-0.003 (0.028)	1.00 [0.94-1.05]	1	-2.54	-0.001	0.827	0.927
IL-24	Allergic rhinitis	19	1.00E-05	0.005 (0.051)	1.00 [0.91-1.11]	0.476	-0.017	-0.016	0.175	0.928
TNFSF14	Allergic rhinitis	37	1.00E-05	0.003 (0.029)	1.00 [0.95-1.06]	0.793	-0.245	0.006	0.295	0.932
CCL8	Allergic rhinitis	46	1.00E-05	-0.002 (0.020)	1.00 [0.96-1.04]	0.148	0.18	-0.003	0.541	0.938
IL-2	Allergic rhinitis	22	1.00E-05	-0.004 (0.057)	1.00 [0.89-1.12]	0.034	0.388	-0.01	0.504	0.948
IL-33	Allergic rhinitis	23	1.00E-05	0.003 (0.043)	1.00 [0.92-1.09]	0.377	0.062	0.012	0.225	0.951
TNFRSF9	Allergic rhinitis	35	1.00E-05	0.003 (0.047)	1.00 [0.91-1.10]	7.07E-05	0.545	0.006	0.578	0.955
CX3CL1	Allergic rhinitis	31	1.00E-05	0.002 (0.054)	1.00 [0.90-1.11]	5.78E-03	0.435	0.022	0.078	0.966
ARTN	Allergic rhinitis	31	1.00E-05	0.000 (0.037)	1.00 [0.93-1.07]	0.759	-0.236	0.011	0.17	0.991
